# Factors associated with drinking and being satisfied with tap water in Indigenous communities in Saskatchewan, Canada

**DOI:** 10.1080/22423982.2018.1466605

**Published:** 2018-04-26

**Authors:** Silvia Bermedo-Carrasco, Lalita Bharadwaj, Cheryl L. Waldner

**Affiliations:** a School of Public Health, University of Saskatchewan, Saskatoon, SK, Canada; b Western College of Veterinary Medicine, University of Saskatchewan, Saskatoon, SK, Canada

**Keywords:** Drinking water, tap water satisfaction, Indigenous, Saskatchewan, tap water quality

## Abstract

Previous studies have described concerns regarding tap water in Indigenous communities, yet there is little information on participants who report drinking their tap water and being satisfied with its quality. This study undertaken with members of 8 Indigenous communities in Saskatchewan, Canada, and identified factors associated with both the decision to drink tap water at home and being satisfied with its quality. We examined the importance of factors such as individual attributes, experiences, attitudes, household and community-based variables. Less than one-quarter of participants (23.4%) drank tap water and were satisfied with its quality. Individuals who did not boil tap water (odds ratio [OR] = 5.76, 95% confidence interval [CI] = 1.68–19.8), those who did not experience tap water odour (OR = 2.38, 95% CI = 1.26–4.50) and participants living in communities away from urban centres (OR = 2.74, 95% CI = 1.63–4.51) were more likely to drink and be satisfied with their tap water. Concerns about the environment had the most impact on community members aged 55+ years. Those not reporting concerns about environmental problems affecting water (OR = 11.4, 95% CI = 3.10–42.2) were much more likely to drink and be satisfied with their tap water. Programmes to improve water quality, reduce the need for boil water advisories and increase community confidence in the environment could improve tap water satisfaction and consumption.

## Introduction

Tap water is the primary source of drinking water in Canada [1]. Most Canadian households (88.9%) are served by piped water systems, while 11.1% of families rely on private wells or truck-delivered water [2]. Consumption of tap water has been associated with a number of different factors including tap water taste, its odour and its physical characteristics [3]. A recent survey found that 89% of Canadians were confident or somewhat confident with the quality and safety of tap water at home [4].

Although most people in Canada enjoy the benefits of safe reliable drinking water, 1% of the population, primarily from rural and Indigenous communities, have no access to adequate quality tap water and sanitation systems [5,6]. Compared to other Canadians, Indigenous homes are 90 times more likely to be without running water [7]. Inadequate access to safe drinking water is an ongoing problem in many Indigenous communities in Canada [8] and is another example of the health inequities faced by Indigenous peoples [9]. Gastrointestinal and skin problems have also been described in association with poor drinking water quality in Indigenous communities in Canada [10]. A recent study in western Canada identified that Indigenous community members who reported rarely or never drinking their tap water and who were dissatisfied with their tap water were also more likely to self-report health effects from drinking water [11].

In 2001, Indian and Northern Affairs Canada reported that the quality and safety of three-quarters of the water systems in Indigenous communities were at high risk [8]. Regardless of the numerous efforts to overcome water-related issues [8,12], the quality of water in 73% of the water systems in Indigenous communities in Canada continued to be classified as medium or high risk for human health in 2011 [12]. Water systems were described as being of limited quality in 2011 in 72% of the 70 Indigenous communities in the province of Saskatchewan [12].

Studies among Indigenous communities across Canada have found limited access to safe drinking water as a barrier to health [10] and low tap water consumption among community members who were concerned about health-related issues [13]. Additionally, in studies assessing tap water consumption in Indigenous communities, it is not always clear whether respondents who did report drinking tap water were actually satisfied with its quality or whether they drank tap water because there was no other accessible option. The objectives of this study were to describe the proportion of individuals from Saskatchewan Indigenous communities who drink tap water and are satisfied with their tap water quality and then to identify factors associated with reporting both drinking tap water and being satisfied with its quality.

## Methods

### Study design and description of the participating communities

A community-based participatory approach was applied to the cross-sectional study design, questionnaire development and data collection. Partnerships between 8 Indigenous communities and the researchers formed the starting point for this study and are documented in a report examining self-reported health status [11].

The total on-reserve population from the 8 communities was 7,132 residents. Participating communities varied in size from ≤500 to >7,000 persons (median = 823 people). Most communities (6/8) were affiliated with a tribal council, and 5 of 8 communities were closer than 70 km to an urban centre and had an independent source of revenue [11].

Of the 8 water treatment systems, 2 were a combination of greensand and reverse osmosis, 2 were manganese greensand only, 1 was a combination of biological filtration and reverse osmosis, 1 was rapid sand and 1 community received treated water piped from a provincially regulated system off-reserve. The final community had 2 water treatment systems with manganese greensand and reverse osmosis [11]. Manganese greensand water treatment was used in 5 of 8 communities. In 4 communities, the water system output was ≤400 m^3^/day and water plants were ≥20 years old [11].

Both piped and trucked delivery of water was reported by 7 communities and truck only in 1 community. In 2 communities, private wells were the primary water source for some homes. Outdoor cisterns were used in 7 of 8 communities and indoor tanks for water storage in 1 community [11].

### Data collection

Community members were trained and employed to administer paper-based questionnaires in each of the participating communities. Community research administrators were asked to complete 1 survey per household with interested residents aged 18+ years. The questionnaire used for data collection included both closed- and open-ended questions. Most questionnaires were filled out during face-to-face meetings. Data were collected between June 2010 and November 2014.

Paper-based questionnaires were stored at the University of Saskatchewan. Survey responses were entered into database software (Microsoft Access). Participating Indigenous communities shared the community-level data reported in this study through e-mail or telephone. The study was approved by the Behavioural Research Ethics Board of the University of Saskatchewan (Beh# 1–96). Verbal consent was given to the community research administrators before beginning the questionnaire.

### Outcome and potential risk factors

The outcome of interest in this study was based on 2 survey questions. The first question was whether respondents always drank water straight from their tap. The second question enquired whether participants were somewhat or very satisfied with the quality of the tap water at home. Respondents who self-declared both always drinking water straight from the tap and being satisfied with the quality of tap water were grouped as “Yes, drink tap water and satisfied with tap water quality”; otherwise, they were grouped as “No, do not drink tap water or drink tap water and unsatisfied”.

**Figure 1.**
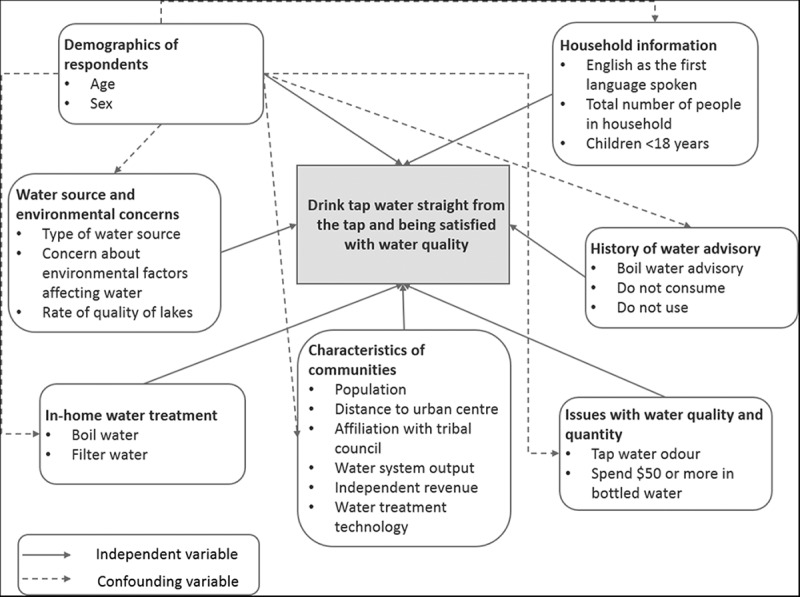
Causal diagram depicting the hypothesised relationships among potential risk factors.

Potential factors which were associated with the decision to drink tap water and be satisfied with its quality considered for this analysis included: individual demographic information, experiences, attitudes and household variables, as well as community-based factors (Table 1). Figure 1 depicts a causal diagram showing the hypothesised relationships among potential risk factors.

**Table 1.** Individual, household and community factors evaluated for their association with drinking water straight from the tap and being satisfied with its quality in 8 Indigenous communities in Saskatchewan (n = 542).

	Total frequency^a^	Drank tap water and were satisfied?					
	n(%)	Yes(%)	No(%)	UOR^b^	95% CI^c^		p-Value
*Demographic and household information*							
Sex of person completing survey							
Male	187(38.9)	44(23.5)	143(76.5)	1.11	0.72	1.71	0.64
Female	294(61.1)	75(25.5)	219(74.5)	Ref.			
Age of person completing survey							
18–34	138(28.3)	37(26.8)	101(73.2)	1.01	0.56	1.82	0.96
35–54	253(52.0)	59(23.3)	194(76.7)	0.90	0.53	1.54	0.71
55+	96(19.7)	24(25.0)	72(75.0)	Ref.			
Language first spoken was English							
Yes	355(69.6)	79(22.3)	276(77.7)	1.00	0.61	1.62	0.99
No	155(30.4)	37(23.9)	118(76.1)	Ref.			
Total number of people in household							
6+	171(32.9)	44(25.7)	127(74.3)	1.17	0.76	1.78	0.48
1–5	349(67.1)	80(22.9)	269(77.1)	Ref.			
Children aged 0–5 years in household							
Yes	233(43.0)	53(22.8)	180(77.2)	0.90	0.60	1.33	0.59
No	309(57.0)	74(24.0)	235(76.0)	Ref.			
Children aged 6–17 years in household							
Yes	286(52.8)	75(26.2)	211(73.8)	1.37	0.92	2.05	0.12
No	256(47.2)	52(20.3)	204(79.7)	Ref.			
*Water source and environmental concerns*							
Surface water							
Yes	168(32.5)	39(23.2)	129(76.8)	1.11	0.56	2.22	0.77
No	349(67.5)	83(23.8)	266(76.2)	Ref.			
Ground water							
Yes	341(66.0)	82(24.1)	259(75.9)	0.90	0.45	1.80	0.76
No	176(34.0)	40(22.7)	136(77.3)	Ref.			
Concern about environmental factors affecting water quality							
Yes	169(37.0)	30(17.8)	139(82.2)	0.43	0.26	0.70	0.001
No	288(63.0)	86(29.9)	202(70.1)	Ref.			
Rate quality of lakes, streams and rivers							
Okay or good	273(56.4)	70(25.6)	203(74.4)	1.89	1.19	2.99	0.01
Poor	211(43.6)	39(18.5)	172(81.5)	Ref.			
*In-home water treatment*							
Boil water							
Always or most of the time	69(13.0)	4(5.8)	65(94.2)	0.19	0.07	0.53	0.001
Rarely or never	463(87.0)	122(26.4)	341(73.6)	Ref.			
Filter tap water							
Always or most of the time	45(8.5)	7(15.6)	38(84.4)	0.60	0.26	1.34	0.21
Rarely or never	487(91.5)	118(24.2)	369(75.8)	Ref.			
*History of water advisories*							
Ever experienced boil water advisory							
Yes	297(57.3)	75(25.2)	222(74.8)	1.24	0.81	1.90	0.32
No	221(42.7)	45(20.4)	176(79.6)	Ref.			
Ever experienced do not consume advisory							
Yes	123(23.7)	25(20.3)	98(79.7)	0.81	0.48	1.39	0.45
No	395(76.3)	95(24.1)	300(75.9)	Ref.			
Ever experienced do not use advisory							
Yes	45(8.7)	8(17.8)	37(82.2)	0.72	0.33	1.57	0.41
No	473(91.3)	112(23.7)	361(76.3)	Ref.			
*Issues with water quality and quantity*							
Ever experienced tap water odour							
Yes	121(23.4)	18(14.9)	103(85.1)	0.49	0.28	0.87	0.01
No	397(76.6)	102(25.7)	295(74.3)	Ref.			
Household bottled water cost ≥$50 per month							
Yes	51(9.7)	5(9.8)	46(90.2)	0.35	0.14	0.89	0.03
No	476(90.3)	119(25.0)	357(75.0)	Ref.			
*Community characteristics*							
Population size on reserve							
Above median for participating communities	308(56.8)	85(27.6)	223(72.4)	1.76	0.85	3.66	0.13
Below median for participating communities	234(43.2)	42(18.0)	192(82.0)	Ref.			
Affiliation with tribal council							
Yes	397(73.2)	90(22.7)	307(77.3)	0.72	0.29	1.77	0.48
No	145(26.8)	37(25.5)	108(74.5)	Ref.			
Distance to urban centre							
≥70 km	188(34.7)	63(33.5)	125(66.5)	2.19	1.18	4.09	0.01
<70 km	354(65.3)	64(18.1)	290(81.9)	Ref.			
Water system output							
>400 m^3^/day	257(47.4)	52(20.2)	205(79.8)	0.78	0.35	1.70	0.53
≤400 m^3^/day	285(52.6)	75(26.3)	210(73.7)	Ref.			
Independent source of revenue							
Yes	353(65.1)	85(24.1)	268(75.9)	1.31	0.45	2.37	0.93
No	189(34.9)	42(22.2)	147(77.8)	Ref.			
Manganese greensand plant to treat water							
Yes	293(54.1)	55(18.8)	238(81.2)	0.60	0.28	1.25	0.17
No	249(45.9)	72(28.9)	177(71.1)	Ref.			
Plant age							
≥20 years	247(45.6)	59(23.9)	188(76.1)	1.20	0.54	2.69	0.66
<20 years	295(54.4)	68(23.0)	227(77.0)	Ref.			

^a^ The total number of individuals answering each specific question. Note this does not always add up to 542 as participants had the option to not answer individual questions.

^b^ UOR: unconditional or unadjusted odds ratio accounting for community as a random effect.

^c^ 95% CI: 95% confidence intervals.

### Statistical analysis

Descriptive statistics were reported for the outcome of interest and each of the potential risk factors. We used a 3-step method to estimate the questionnaire response rate specifically for the outcome of interest. In step 1, we computed the number of individuals per community who answered the 2 questions regarding whether they drank their tap water and were satisfied. Then, we divided the population size in each community by the average number of individuals per household to obtain the number of households per community. Finally, we estimated the response rate using the number of respondents per community as the numerator and the number of households per community as the denominator.

Before proceeding with the primary study analysis, the attributes of respondents who provided complete information regarding the outcome of interest were summarised and compared to those who did not answer both of the component questions making up the outcome variable. To compare the 2 groups, associations between each attribute of participants and having or not having complete outcome information were assessed using a 2-level generalised linear mixed model for a binomial outcome with a logit link function to account for the hierarchical structure of the data (household responses within community). Community was considered as a random intercept to account for clustering of household responses within location in both this and the model examining the primary study objective.

Prior to building the 2-level multivariable model for the primary study objective, an intraclass correlation coefficient was computed from the null model or intercept-only model to evaluate the magnitude of potential clustering of the outcome responses within communities [14]. Unconditional (or unadjusted) associations were then evaluated between each of the potential risk factors of interest and the study outcome using all of the available observations for each risk factor. Risk factors with a p-value ≤0.2 were initially retained [14]. A manual backward stepwise selection strategy was then used to build the main effects model, retaining factors with p-values ≤0.05. A type 3 likelihood ratio test was used to assess variables with more than 2 categories.

Factors with p-values >0.05 in the final model were further evaluated as potential confounding variables, based on a ≥20% change in the coefficients for the other risk factors and the causal diagram. The presence of contextual effects was examined by considering the proportion of respondents by community who answered affirmatively to each of the risk factors included in the final model, except for sex and age. Biologically plausible interactions were explored between factors retained in the final model and age and then sex. Population-averaged odds ratios (ORs) were computed by adjusting the subject-specific coefficients generated by the final model [14]. Observations with missing values were excluded for variables contained in the final model.

The fit and adequacy of the final model were evaluated by plotting residuals and calculating the area under the receiver operator characteristic (ROC) curve. Stata 13 (StataCorp LP, College Station, TX, USA) was used to complete the statistical analysis.

## Results

### Descriptive statistics

Out of 590 participants from the 8 Indigenous communities, 542 provided complete information for the 2 survey questions used to build the study outcome. The median response rate for these questions was 35.5% (range = 14.5–56.7%) of all households in the 8 Indigenous communities. The mean and the median numbers of participants per community with complete outcome information were 68 (SD = 22) and 71, respectively. In total, 48 household surveys did not include complete information for the study outcome of interest.

Of the total 542 community members answering the questions regarding drinking tap water and tap water satisfaction, 35.2% (191/542) always drank water straight from the tap regardless of being satisfied with its quality, while 45.8% (248/542) were satisfied with tap water quality irrespective of their tap water consumption. Almost one-quarter or 23.4% (127/542) of study participants drank water directly from the tap while at home and were satisfied with its quality. However, 11.8% (64/542) drank tap water but were not satisfied with its quality.

Table 1 summarises all responses from the 542 surveys where outcome data were complete, as well as individual and household characteristics of participants according to whether or not they drank tap water and were satisfied. Completeness of data provided on these surveys for other factors of interest varied from a minimum of 77.5% for “total number of people in household” to 100% for “children aged 0–5 years in household”. Most community members completing the household questionnaire were women aged 35–54 years, described their primary language at home as English, had 1–5 family members living in the household and resided in households with children aged 6–17 years.

There were no significant differences in the distribution of factors reported on surveys from households providing complete outcome information (n = 542, Table 1) and those households where survey data for the outcome variable was incomplete (n = 48) (data not shown). All subsequent results are limited to those surveys with complete outcome data (n = 542).

#### Water source, in-home water treatment and issues with water quality and quantity

Most respondents described having ground water as the source of tap water at home (Table 1) as 6 of 8 communities had a ground water source. In-home tap water treatment, either boiling or filtering, was uncommon among survey respondents (Table 1). Most participants reported never having experienced tap water odour and stated not being concerned about environmental factors affecting water quality (Table 1). When asked about water quality, most individuals rated quality of lakes, streams and rivers as “okay or good” and reported spending <$50 per month on bottled water (Table 1).

#### History of water advisories

Community members were also asked whether they had experienced water advisories. Most respondents (57.3%) reported having experienced a “boil-water” advisory, while 23.7% and 8.7% had experienced either a “do not consume” or a “do not use” tap water advisory, respectively (Table 1).

#### Reported reasons for choosing to drink tap water at home

Table 2 summarises the main reasons for choosing to drink tap water at home as described by respondents who drank their tap water and were satisfied. Of these participants, most reported that convenience and availability of tap water were factors related to choosing tap water.

**Table 2.** Most common reasons for choosing to drink tap water provided by participants who drank tap water and were satisfied with water quality in 8 Indigenous communities (n = 124)^a^.

	Total respondents	%
Convenience of tap water	67	54.0
Tap water is more available	62	50.0
No difference/just as good as bottled water	56	45.2
Cost of bottled water	28	22.6
Tap water tastes better	16	12.9
Make with juice, etc., to disguise taste	16	12.9
Tap water is safer	14	11.3
Plastic water bottles impact environment negatively	13	10.5
Tap water is better for health	11	8.9
Do not know	10	8.1
Do not trust quality of bottled water	8	6.5
Do not like to drink out of plastic bottles	5	4.0
Have a filter for tap water	3	2.4

^a^Three of the 127 respondents who drank their tap water and were satisfied did not answer this question.

### Factors associated with drinking straight tap water and being satisfied with tap water quality

In the null or intercept-only multilevel model, the intraclass correlation coefficient or the proportion of variation in the outcome data explained by differences among communities was 7.5% (likelihood ratio test, p* = *0.0001).

The unconditional analysis identified 9 potential factors associated with the outcome of interest (Table 1), including the presence of children aged 6–17 years (p* = *0.12); having concerns about environmental factors affecting water quality (p* = *0.001); the quality of lakes, streams and rivers (p* = *0.01); boiling water at home (p* = *0.001); having ever experienced tap water odour (p* = *0.01); spending ≥$50 in bottled water (p* = *0.03); the population size on reserve (p* = *0.13); distance to urban centres (p* = *0.01) and having access to water treated in a manganese greensand plant (p* = *0.17). These factors were retained for further consideration in the multilevel multivariable analysis.

During the model building, population size on reserve (p* = *0.65), the presence of children aged 6–17 years (p* = *0.39), having access to water treated in a manganese greensand plant (p* = *0.34) and spending ≥$50 per month in bottled water (p* = *0.05) were initially removed from the model due to non-significance and then later tested as potential confounders. However, none of these factors were found to be confounders of the coefficients of the other variables retained in the model. Age and sex were included in the final model based on potential differences in opinions and perceptions about water based on age and between men and women. There were no significant contextual effects, and no interactions with sex were identified in the final model.

From the multivariable model summarised in Table 3, individuals who rarely or never boiled water (OR = 5.76), participants who did not experience tap water odour (OR = 2.38) and those whose communities were farthest away from urban centres (OR = 2.74) were more likely to drink water straight from the tap and be satisfied with the quality of tap water.

**Table 3.** Factors associated with drinking tap water straight from the tap and being satisfied with its quality in the final multivariable model in 8 Indigenous communities.

	OR	95% CI		p-Value
Boil water				
Rarely or never	5.76	1.68	19.8	0.01
Always or most of the time	Ref.			
Ever experienced tap water odour				
No	2.38	1.26	4.50	0.01
Yes	Ref.			
Distance to urban centre				
≥70 km	2.74	1.63	4.61	<0.001
<70 km	Ref.			
Sex of person filling survey				
Male	1.41	0.85	2.34	0.19
Female	Ref.			
Interaction between *age* and *concern about environmental factors affecting tap water quality*				0.03

Results are reported as population-averaged odds ratios (ORs), 95% confidence intervals (95% CIs) and their corresponding p-values (p) (n = 381).

A significant interaction was also detected between “having concerns about environmental factors affecting water quality” and age (p* = *0.03). *Post hoc* comparisons identified differences in the likelihood of drinking and being satisfied with household tap water between being and not being concerned about environmental issues affecting water according to age groups (Table 4).

**Table 4.** Population-averaged odds ratios (ORs), 95% confidence intervals (95% CI) and p-values (p) for factors for drinking tap water and being satisfied with tap water quality in the final multivariable model.

Comparisons	OR	95% CI		p-Value
Between non-concerned and concerned about environmental factors affecting water quality				
18–34 vs. 18–34 years	1.57	0.57	4.34	0.38
35–54 vs. 35–54 years	1.70	0.83	3.45	0.15
55+ vs. 55+ years	11.4	3.10	42.2	<0.0001
Within non-concerned about environmental factors affecting water quality				
35–54 vs. 18–34 years	1.24	0.64	2.40	0.52
55+ vs. 18–34 years	2.92	1.25	6.82	0.01
55+ vs. 35–54 years	2.35	1.07	5.18	0.03
Within concerned about environmental factors affecting water quality				
35–54 vs. 18–34 years	1.15	0.40	3.29	0.79
55+ vs. 18–34 years	0.40	0.10	1.63	0.20
55+ vs. 35–54 years	0.35	0.10	1.18	0.09

Variables reported here include the interaction between “having concerns about environmental issues affecting water” and age groups (n = 381).

Individuals who were not concerned about environmental issues affecting water were more likely than those who were concerned to drink and be satisfied with tap water, but only in the oldest age group (OR = 11.4). Similarly, among non-concerned, those in the oldest age group were 2.92 and 2.35 times more likely to drink tap water and be satisfied with its quality when compared to individuals aged 18–34 and 35–54 years, respectively. No age-related differences in the likelihood of drinking and being satisfied with household tap water were identified within individuals concerned about environmental impacts on water quality. The area under the ROC curve for the final model was 0.69 (95% confidence interval [CI] = 0.63–0.74).

## Discussion

For Indigenous people, access to water is part of wellness, a concept rooted on the balance among different components that connect individuals with Creation [15]. Although clean safe water has an important spiritual role for Indigenous communities, as well as functions in health, nutrition, the necessities of daily living and economics, access to reliable drinking water is not a reality in many of these communities across Canada [7,8].

We found that 35% of participants drank their tap water, regardless of being satisfied with its quality. However, less than one-quarter of participants drank water straight from the tap at home and were satisfied with the quality of tap water they did drink. Despite the lower reported prevalence of tap water consumption in our study compared with previous Canadian reports from Indigenous and other rural communities [16,17], the satisfaction with tap water in our sample was similar to that in a study among Indigenous communities in Canada [16]. Improvements in on-reserve water quality and access to more information about water testing have led to increasing levels of trust in water quality among Indigenous residents who drink tap water [16]. A study in rural Saskatchewan found that living in the same area for more than 10 years and not experiencing water advisories were factors associated with daily tap water consumption [17].

Other studies have found that tap water consumption can be influenced by its organoleptic properties, health concerns, risk perception, access to other water sources, attitudes towards water chemicals, trust of water suppliers, past problems with drinking water, as well as information provided by the media and interpersonal sources of information [3,18]. Past experiences with boil water advisories, trust in water supply, culture and gender have also been reported to influence the perception of drinking water quality [19–22]. In our sample, community residents reported convenience and availability of tap water as common reasons to choose drinking tap water. However, when examining the associations between various community and individual attributes, we identified several other factors associated with increased likelihood of tap water consumption and satisfaction. These included the absence of concerns regarding environmental factors, older age, increasing distance from urban centres, not boiling water before use and a lack of odour.

The perception of water quality in Indigenous communities in Canada is reinforced by the frequency of drinking water advisories and the presence of contaminants in the water [16]. We explored whether having concerns about environmental issues affecting water was associated with the likelihood of drinking tap water that was considered satisfactory. Among individuals without environmental concerns, we found that those aged 55+ were more likely to drink and be more satisfied with tap water than their younger counterparts. A survey conducted with residents after discovery of contamination in their local water supply found the degree of concern to be less among long-term or older residents compared to women with young children [23]. Results could suggest that community members with long-term residence in the community have higher confidence in their water in the absence of specific environmental issues. The same age-related differences in the likelihood of drinking and being satisfied with the water were not apparent among those who did have environmental concerns in the present study.

Other Canadian studies about self-reported effects of tap water on health among Indigenous communities also found differences by age groups [11,13]. Dupont et al. [13] described that younger Indigenous individuals were more likely than those aged 55+ years to associate tap water with becoming sick. A recent study in Saskatchewan described that the difference in the likelihood of self-reporting health effects associated with tap water was greater among young individuals compared to participants aged 55+ years, especially among those who were dissatisfied with tap water [11]. The authors hypothesised that the perception of drinking water could be impacted by the greater access to information that young people have in comparison to older individuals [11].

In the present study, there was a significant difference in the likelihood of drinking and being satisfied with tap water only for those who were greater than 55 years of age. The absence of a significant difference in the younger age groups was in part due to the much lower proportion of individuals who drank and were satisfied with their tap water in the absence of reported environmental concerns. That is, fewer young people drank tap water limiting the power to detect a difference. Differences may also exist among young and older respondents access to information and their level of awareness, understanding and acceptance of environmental influences on water quality. Differences in levels of awareness could affect beliefs of young people regarding tap water and, potentially, their own health. Awareness of environmental issues in youth has been associated with having scientific knowledge about environmental problems [24]. However, increased levels of knowledge might not translate into having a pro-environment behaviour [25], despite the greater access of Indigenous youth to the Internet compared to older adults [26].

Absence of concerns about negative health effects related to drinking tap water could be another factor explaining differences in tap water consumption and tap water satisfaction observed in our study between young and old community members not concerned about environmental threats. In a previous analysis of data shared by these communities [11], compared to younger individuals, older participants were less likely to self-report health effects associated with tap water, especially among those who were more satisfied with tap water. This study also identified that participants not concerned about environmental problems affecting water were less likely to self-report health effects believed to be associated with tap water [11].

Despite water system infrastructure challenges reported in Indigenous communities [8,12,27–29], we found that individuals living in communities farther away from urban centres were more likely to drink and be satisfied with their tap water. This finding could reflect the high value that participants posed to having tap water available in their homes. Being satisfied with tap water in distant communities could also be related to the cost of having access to alternative sources of drinking water. Bottled water is typically more expensive than tap water in most regions in Canada [30]. In remote Saskatchewan Indigenous communities, the price of bottled water could be higher than the average national and provincial cost due to limited access to retail suppliers or the additional costs associated with transportation [31].

In this study, individuals who reported not boiling their tap water (87%) were more likely to drink and be satisfied with it. This finding was not surprising, given changes in water taste associated with boiled water [32]. Additionally, filtering and boiling are associated costs to tap water consumption that could have discouraged tap water use in our sample. Organoleptic properties of drinking water, odour and taste have also been identified as important factors related to the perception of water quality [3,18]. Our results indicated that not having experienced water odour in the past increased the probability of drinking and being satisfied with tap water.

In the present study, individuals who were 55+ years and who had no reported concerns about the environment and from communities farthest from urban centres were most likely to drink their tap water and be satisfied with its quality. Perceptions around water quality and safety have a strong impact on decisions about tap water consumption. Trust in drinking water providers and control of drinking water provision are key factors to address to improve consumption. Locally organised training and educational programmes may be a way forward to improve trust and ultimately community tap water consumption.

Several Indigenous communities, through the development of water operator training programmes, are tackling issues of trust and control by empowering communities to manage their own drinking water systems. The Safe Water Project [33], envisioned by the Chiefs of Keewaytinook Okimakanak, is just one example of a novel Indigenous communities run training programme aimed at improving drinking water provision. Instilling pride and confidence in water systems managed by Indigenous communities could lead to improved tap water consumption and satisfaction. The establishment of the Ontario First Nations Technical Services Corporation in 1995 is another example of fostering trust in community drinking water through the building of technical self-reliance and water treatment capacity [34]. The adoption of an Indigenous community-owned full-service-decentralised water authority may be another possibility to improve trust and confidence in drinking water management in communities [35]. Development of Indigenous technical cooperatives and water authorities may be a way forward to improved drinking water consumption and satisfaction.

To our knowledge, this is the first positive deviation study conducted in Canada exploring factors associated with whether residents of Indigenous communities drink and are satisfied with their tap water. Insights into perceptions and concerns about tap water quality with these communities could lead to improvements in water management and monitoring, water supply services and communications of and education about water-related risks. Others have demonstrated research studies with Indigenous communities can inform funding applications and policies to improve access to safe drinking water [36]. The community-based participatory research (CBPR) method used to connect with the communities, design questions and gather data is a strength of this study [11]. This approach enhanced trust between communities and the researchers and led to further research collaborations in some communities. While there are many critical advantages of using a CBPR, this methodology can substantially increase time to complete research studies [11].

Limitations to this study include its cross-sectional design. It was not possible to determine cause and effect between the dependent variable and the risk factors of interest. We did not have a means of measuring how much water individuals consumed leading to the potential for misclassification. Data provided by participants were potentially subject to recall bias and social desirability. The power of this study was somewhat limited, given the impact of the proportion of community households approached to participate and missing information from incomplete surveys. The findings of this study are most applicable to communities with ground water sources, as there were only 2 communities that relied on surface water.

In conclusion, using a CBPR approach, we identified that less than a quarter of participants living in 8 Indigenous communities in Saskatchewan drank water straight from the tap and were satisfied with tap water quality. We identified a high probability of drinking and being satisfied with tap water among participants aged 55+ years who were not concerned about environmental issues affecting water. Living away from urban centres was associated with being more likely to be satisfied with tap water. Among participants who drank tap water, those who did not boil tap water and individuals who did not experience tap water odour were also more likely to be satisfied with tap water quality. Results of this study provide understanding of tap water consumption and tap water satisfaction among Indigenous communities in western Canada. Our findings could translate into programmes to improve water quality, reduce boil water advisories and upgrade community confidence in the environment that further increase tap water satisfaction and consumption, especially among young individuals.

## Geolocation information

This study was conducted in the province of Saskatchewan, Canada. Specific information about participating communities cannot be provided due to confidentiality requirements.
